# Development of ERN together with an internal model of audio-motor associations

**DOI:** 10.3389/fnhum.2013.00471

**Published:** 2013-09-03

**Authors:** Kai Lutz, Roman Puorger, Marcus Cheetham, Lutz Jancke

**Affiliations:** ^1^Department of Neuropsychology, Institute of Psychology, University of ZürichZürich, Switzerland; ^2^cereneo, Center for Neurology and RehabilitationVitznau, Switzerland

**Keywords:** error processing, EEG, internal model, error related negativity, piano, tool transformation

## Abstract

The brain's reactions to error are manifested in several event related potentials (ERP) components, derived from electroencephalographic (EEG) signals. Although these components have been known for decades, their interpretation is still controversial. A current hypothesis (first indicator hypothesis) claims that the first indication of an action being erroneous leads to a negative deflection of the EEG signal over frontal midline areas. In some cases this requires sensory feedback in the form of knowledge of results (KR). If KR is given, then the first negative deflection can be found around 250 ms after feedback presentation (feedback-related negativity, FRN). When KR is not required, a negative deflection is found already around 100 ms after action onset (ERN). This deflection may be evoked when a mismatch between required and actually executed actions is detected. To detect such a mismatch, however, necessitates knowledge about which action is required. To test this assumption, the current study monitored EEG error components during acquisition of an internal model, i.e., acquisition of the knowledge of which actions are needed to reach certain goals. Actions consisted of finger presses on a piano keyboard and goals were tones of a certain pitch to be generated, thus the internal model represented audio-motor mapping. Results show that with increasing proficiency in mapping goals to appropriate actions, the amplitude of the ERN increased, whereas the amplitude of the FRN remained unchanged. Thus, when knowledge is present about which action is required, this supports generation of an ERN around 100 ms, likely by detecting a mismatch between required and performed actions. This is in accordance with the first indicator hypothesis. The present study furthermore lends support to the notion that FRN mainly relies on comparison of sensory targets with sensory feedback.

## Introduction

Error processing during goal-oriented tasks is associated with a series of ERP components. One error component is the classical *error negativity* (Ne; Falkenstein et al., 1991), or *error-related negativity* (ERN; Gehring et al., 1993) that evolves at 40–50 ms shortly after the motor response begins and peaks at around 100 ms. Of all components engaged in error processing, the ERN has received most attention. It is associated with errors in a variety of tasks (Krigolson and Holroyd, 2007) and can be elicited by various error signals and mediated by different neuronal systems (Krigolson and Holroyd, 2007; Holroyd et al., 2009; Ham et al., 2013). Prominent among the associated brain areas are the medial prefrontal cortex (mPFC) and especially the posterior frontomedial cortex (pFMC; including the anterior cingulate cortex, ACC), having been implicated in error-monitoring in different domains and with different temporal dynamics. But other brain regions interact with the mPFC during error-related processing, notably the insular cortex (Donamayor et al., 2012) and other structures associated with affect such as the dopaminergic midbrain structures (Holroyd and Coles, 2002).

According to Coles et al. (2001), the ERN is evoked by a mismatch between required action and actually performed action. An ERN is evoked if, for example, it becomes evident during initiation or performance of an action that the chosen action will not lead to the desired results. But there is still some debate whether ERN reflects a specific aspect of error detection processing (e.g., the initial error signal, Stahl, 2010) or is associated with error probability (Holroyd and Coles, 2002), conflict monitoring (Yeung et al., 2004) or with the processing of other aspects of primary task performance like decision evidence and uncertainty (Steinhauser and Yeung, 2010).

In highly overlearned movements like speech production (Moller et al., 2007), and piano playing by well-trained pianists (Ruiz et al., 2009) overt erroneous actions are preceded in 20–70 ms by a negative ERP deflection, even in the absence of auditory feedback (pre-ERN; Ruiz et al., 2009). This error signal may reflect the outcome of forward control processes underlying motor prediction.

A further negative deflection in the ERP, the *feedback-related negativity* (FRN), occurs at around 200–300 ms (Miltner et al., 1997; Luu et al., 2003; Müller et al., 2005). This ERP occurs upon provision of feedback about correctness or precision (knowledge of results, or knowledge of performance, respectively). In the context of musical performance, Maidhof et al. (2010) could further show that a fronto-central negativity around 200 ms after tone onset is evoked in highly trained pianists, hearing unexpected (wrong) notes in a musical piece, and that this negativity is more pronounced if the error appears in response to a keypress, as part of active music production, rather than during passive listening. ERN in a passive listening condition suggests that the respective error component is evoked by (sensory) expectancy violation. Interestingly, in skilled musicians, passive listening to and active production of music, seem to share common processes: the motor system is involved in passive listening and the sensory system is involved in playing an instrument without hearing the tones (Baumann et al., 2005, 2007), thus, it is hard to differentiate between sensory and motor processes leading to electroencephalographic (EEG) deflections in skilled musicians when playing their instrument.

The function of these components is not entirely clear. The fact that an ERN sometimes peaks at around 100 ms after movement onset and sometimes only after feedback presentation led to the formulation of the “first indicator hypothesis” (Stahl, 2010). In short, this states that an ERN evolves at around 100 ms following the initial signal that the performed action is correct or incorrect. In some settings, internal information will be sufficient to ascertain whether an action is erroneous in failing to lead to the intended goal. In other settings, external feedback is required to establish that the action is erroneous (e.g., the agent knows only after performance feedback in knowledge-based tasks whether the given answer was correct or false). Thus, the ERN and the FRN might represent neural responses to a signal that the expected goal of an action will not be or has not been realized. Interestingly, an ERN can evolve as early as 40–50 ms after movement onset and peak around 100 ms. This raises the question whether this short time is sufficiently long to analyze movement consequences. Assuming insufficient time for such analyses, we considered the possibility that the ERN represents a neural response to expectation that the intended action consequence will be missed. To elaborate this point, consider that information about an action being performed feeds to the error detection system without afferent input from sensor organs. Instead of afferent input, efferent signals are sent by the motor system to effector organs and are copied (forming “efference copies”) to various other sites within the central nervous system. Such a distribution of motor information to multiple receptive systems has been suggested, according to concepts of von Holst and Mittelstaedt (1950) and Sperry (1950), to allow for fast integration of feedforward information into motor control processes (see Miall and Wolpert, 1996). This requires a correct internal model that represents the actions required to reach certain goals in specific situations (Wolpert et al., 1995). By mapping actions to goals, this internal model also enables prediction of the outcome of certain actions in specific situations. Detection of mismatch between the action represented in an inverse model to reach a specific goal and the action actually being performed can thus be used to detect errors in the absence of knowledge of results (KR) and even sensory feedback. Heldmann et al. (2008) reasoned in a similar way in relation to a special variant of decision tasks in which correct responses required both correct decisions and a minimum speed of performance. This task meant that a correct decision indicated by pressing the right button was still considered false if the decision response latency was too high. Heldmann et al. found that an ERN (time-locked to the response) would evolve if response latencies were sufficiently long for subjects to realize that their answer was false. But when the response latency was close to the cutoff point at which this realization was unlikely, no ERN was observed—Instead of an ERN, an FRN evolved in response to error feedback. Similarly, Holroyd et al. (2004) used a 2-alternative response mapping task with either random (unlearnable) or fixed (learnable) stimulus-response mappings to examine feedback and errorrelated brain activity, respectively. The reasoning behind this approach was that it would only be possible with fixed stimulus-response mappings for subjects to decide whether a given answer is correct without relying on feedback. Their functional magnetic resonance imaging results show that both kinds of errors activate the same structure, that is, the dorsal anterior cingulate cortex (dACC), but with a different temporal dynamic.

Holroyd and Coles (2002) proposed that a negative deflection is induced in the EEG due to phasic disinhibition of the ACC after occurrence of an unfavorable event. As such an event could signal error, error detection could be expressed in terms of an unexpected outcome of an action, similar to a prediction error in reinforcement learning theory. The reinforcement learning approach to error processing assumes that whenever information indicates that an action does not result in an expected consequence, basal ganglia mediated ACC disinhibition leads to a negative EEG deflection at frontocentral electrodes (Holroyd et al., 2009).

It follows from this assumption, when considered in combination with the preceding understanding of internal models, that if there is a correct internal model allowing an agent to (A) select the appropriate action to reach a certain goal and (B) to correctly predict action consequences, then the agent does not have to rely on external feedback for error detection. Based on this reasoning, we predicted that a negative EEG deflection would evolve earlier when there is an internal model compared with when there is no such model.

Taken together, we hypothesized that an FRN would peak around 250–300 ms after feedback presentation when there is no internal model and that an ERN would peak around 100 ms after action onset once an internal model has been established. If, on the other hand, internal model predictions do not contribute to the development of an ERN, this component should remain stable with or without an established internal model.

To test this, participants were required to find the correct keys on a piano, in order to reproduce notes played to them as auditory goals. Learning how keypresses are mapped to tones of different pitch in this task entails formation of an internal model. We assume that at the beginning of the experiment, errors would only be detected by processing auditory information and by comparing the self-produced tone with the auditory goal. After formation of an internal model, error components would be elicited by additional processes: (A) mismatch between the motor prediction and performed action and (B) mismatch between anticipated and actually observed sensory effect associated with the action. To further distinguish between these two possibilities, we introduced a condition in which a wrong tone would be heard even though performance was correct. This kind of “error” was termed “external error” in accordance with Ullsperger's terminology (Ullsperger and von Cramon, 2003) or “tool error” (TE) in keeping with Kalveram's formulation of tool transformations as being the transformation processes which translate muscular activity into effects on the environment (Kalveram, 2004).

The current experiment was not designed to investigate the influence of response conflict on ERP components. Instead, we kept the number of possible responses constant and did not introduce any response tendencies.

## Methods

### Participants

Twenty-one Participants, who had never taken piano lessons and had no musical training beyond that required in the school curriculum, took part in the study. All were right handed according to self-report. Participants were reimbursed with a minimum of 40 and a maximum of 60 Swiss Francs, depending on their performance in the experiment. The study was approved by the local ethics committee and conducted in accordance with the Declaration of Helsinki (World Medical Association, 2008).

Due to technical problems, the data of one participant was discarded. Another participant did not learn the mapping of piano keys to tones and was thus excluded from further analysis. Thus, data of 19 participants (three male, 16 female), aged 20–33 years (Mean = 25.8; *SD* = 4.1) were analyzed.

### Technical equipment

**Figure 1:**
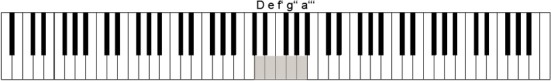
**Layout of the Piano**.

An electronic piano (Yamaha P-60, Yamaha Music Europe, Zürich, Switzerland) was used to play piano tones and record keypresses. Only five keys (d', e', f', g', a') were used, the remaining keys being shielded from view with a cardboard construction. Visual contact with the keys and the hand placed on the keys was prevented by a specially designed plastic screen. The keys used were mapped to the tones D, e, f', g”, a”', thus shifting the original key mapping by −2, −1, 0, 1, and 2 octaves, respectively (see Figure 1). The piano was connected to a PC via a MIDI/Serial converter (Cinetix, Frankfurt/Main, Germany) where in-house software running on Presentation (Neurobehavioal Systems, Albany, CA, USA) recorded time onset and velocity (related to loudness) of the keystrokes and sent MIDI commands to the piano in order to generate tones. Tones were presented to the subject via in-ear headphones (Philips) without sound conduction latencies. EEG was recorded using a Geodesics 128 electrode sensor net while participants were sitting in a sound insulated and electrically shielded EEG chamber on a comfortable chair (RECARO Automotive GmbH, Kirchheim, Germany) equipped with individually adjustable elbow- and chinrests. Tones were additionally transferred outside the chamber to loudspeakers for the experimenter's surveillance.

### Experimental task and procedure

The participants' task consisted of the reproduction of piano tones in the correct pitch. Therefore, one of the five target tones D, e, f', g”, a”' (73.4 Hz, 164.8 Hz, 349.2 Hz, 784.0 Hz, 1760.0 Hz, respectively) was presented for 100 ms at the beginning of each trial. The participants had to select an appropriate key to reproduce the target tone, without knowing, at the beginning, which tones were generated by which key. They had 2 s to respond before the next target tone was presented. Participants were instructed to react at regular intervals, not to leave out any keypresses and to press only one key after each target tone (i.e., not to correct errors).

Trials were presented in blocks of 20, containing each tone four times in pseudo-random order. In order to allow correct finger placement, the middle tone (f') was always presented before each block and was reproduced by the participants without time constraint. This effectively made learning of the mapping of the middle key very easy and led to useful overall learning rates (see results section), revealed by a significant reduction in errors but, at the same time, production of at least 10 trials with an IE per subject at the beginning and at the end of the experiment.

Whenever the subject pressed the correct key to reproduce tone D or a”', with 50% probability (but to a maximum of twice per block), a wrong tone was generated: e instead of D, and g” instead of a”'. This incorrect feedback allowed to separate internal, self-produced errors (IE) from external errors, that is, errors originating in the tool (TE) (Gentsch et al., 2009; Nadig et al., 2010). These two error types can only be distinguished by the subject if an internal model has been established, allowing prediction of action consequences. Thus, with identification of distinct error components for these two error types, the existence of an internal model and its consequence for error processing can be demonstrated.

After each block, participants were asked to estimate how often they had pressed the wrong key. Having participants assess whether they correctly and consciously identified their own errors also gave the participants the opportunity to express any concerns that the tool had malfunctioned (i.e., recognition of tool errors) and thus served as a rough estimate of participants conscious error awareness. After that, participants could start the next block by pressing a button when ready. Ten blocks made up a session, which lasted about 8 min. The whole experiment consisted of 5 sessions. Between the sessions, a short break of about 5 min was inserted in order to check, and, if necessary, restore electrode impedance (see below).

### Analysis of behavioral data

All trials containing one response were included and those with no response or more than one keypress were eliminated from behavioral and electrophysiological analyses. Trials were categorized into the following three classes: (1) correct responses, (2) internal errors (IE: the wrong key was pressed leading to a wrong tone), and (3) tool errors (TE: the correct key was pressed but the piano produced a wrong tone). The total number of IE in sessions 1 and 2 was compared with the total number of IE in sessions 4 and 5 using a paired samples *t*-test to assess whether the number of errors was reduced during the experiment.

### EEG data recording and processing

EEG data were recorded with a sampling rate of 500 Hz. using a Geodesics 128-channel HydroCell Sensor Net connected via NetAmp amplifier to a Mac G4 running NetStation Software (Electrical Geodesics Inc., Eugene, OR, USA). Sensor caps were individually chosen from three sizes to fit the subject's head. The central electrode (#129, Cz) was used as reference for acquisition. During post processing, the signals were re-referenced to the left and right mastoids. Conductivity between Skin and Ag/AgCl electrodes was improved by sponges soaked in KCl solution prior to the experiment. Placement and wetness of electrodes were adjusted in the beginning of the experiment, to make sure that electrical resistance was below 30 kOhm at all electrodes. KCl solution was administered at the sponges during the brakes in between sessions, if resistance had risen above 30 kOhm.

EEG post processing was performed using Brain Vision Analyzer software (Brain Products, München, Germany). Data were digitally filtered using a notch filter at 50 Hz and an infinite impulse response filter with low cutoff frequency of 0.1 Hz and a high cutoff of 30 Hz. Using independent component analysis, 128 sources contributing to the signal were calculated. Artifact sources (eye movement/blinks, and muscle artifacts) were visually identified and eliminated. Subsequently, remaining artifacts were semi automatically identified and removed, using the following criteria: Maximal allowed voltage step: 15 μV/ms, allowed amplitudes between −100 and 100 μV, lowest allowed activity in sliding 100 ms intervals: 0.5 μV. The cleaned signal was divided into segments from 100 ms before to 300 ms after each keypress. Baseline correction (subtracting the average voltage in the interval [−100, 0] ms from each segment) was performed.

Events were aggregated subject-wise into correct responses, IE and TE according to the behavioral analysis. ERPs were calculated for all three classes, separated into the “beginning of the experiment” (blocks 1 and 2) and the “end of the experiment” (blocks 4 and 5). To identify error related components, the correct responses were subtracted from the two error types, resulting in four ERPs: IE(beginning), IE(end), TE(beginning), and TE(end).

Since the most important error components are considered to originate from frontal cortical midline structures, we averaged for the purpose peak detection the signals from electrodes 6, 7, 106, and 129; 6 and 129 represent FCz and Cz, respectively, and 7 and 106 are located ~10 mm lateral from the midpoint between FCz and Cz (128-channel HCGSN layout V1.0, Electrical Geodesics Inc., Eugene, OR, USA). We chose this ROI-based approach based on Ullsperger and von Cramon (2001) in order to cover the medial fronto-central region with better signal to noise ratio. Within these average signals, for each individual, ERN amplitude was identified as the minimum signal from 50 to 150 ms, and FRN amplitude was identified as the minimum signal from 200 to 300 ms.

### Statistics

The peak amplitudes were submitted to a 2 × 2 repeated measures ANOVA (SPSS 19, IBM Deutschland, Ehningen), with the factors “error type” (internal error, and tool error) and “time” (beginning and end of the experiment). To control for violations of sphericity, Greenhouse-Geisser correction was applied. *Post-hoc t*-tests were performed in case of significant main effects or interactions. The ERN and FRN amplitudes were tested separately for significant deviation from zero for IE and TE at the beginning and at the end of the experiment. For each of these conditions, the peak latency of the group mean signal was determined and the signal intensity at this latency was subjected to one sample *t*-tests.

In order to get a more complete picture of the data, we additionally provide topographic representations of the grand average difference signal for both error types during the peri-response time window, split up into 50 ms intervals.

## Results

### Behavioral data

**Figure 2:**
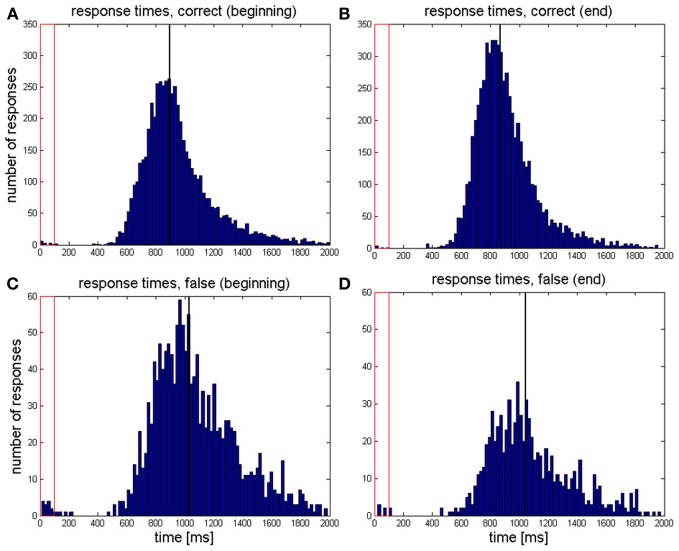
**Distribution of response times pooled over all responses which were (A) correct during the first two sessions, (B) correct during the last two sessions, (C) false during the first two sessions, and (D) false during the last two sessions**. The black vertical line indicates the median of all response times of the respective category. The red rectangle depicts the time period during which the target tone was presented.

The distributions of response times pooled over all correct and incorrect trials at the beginning and at the end of the training are depicted in Figure 2.

**Figure 3:**
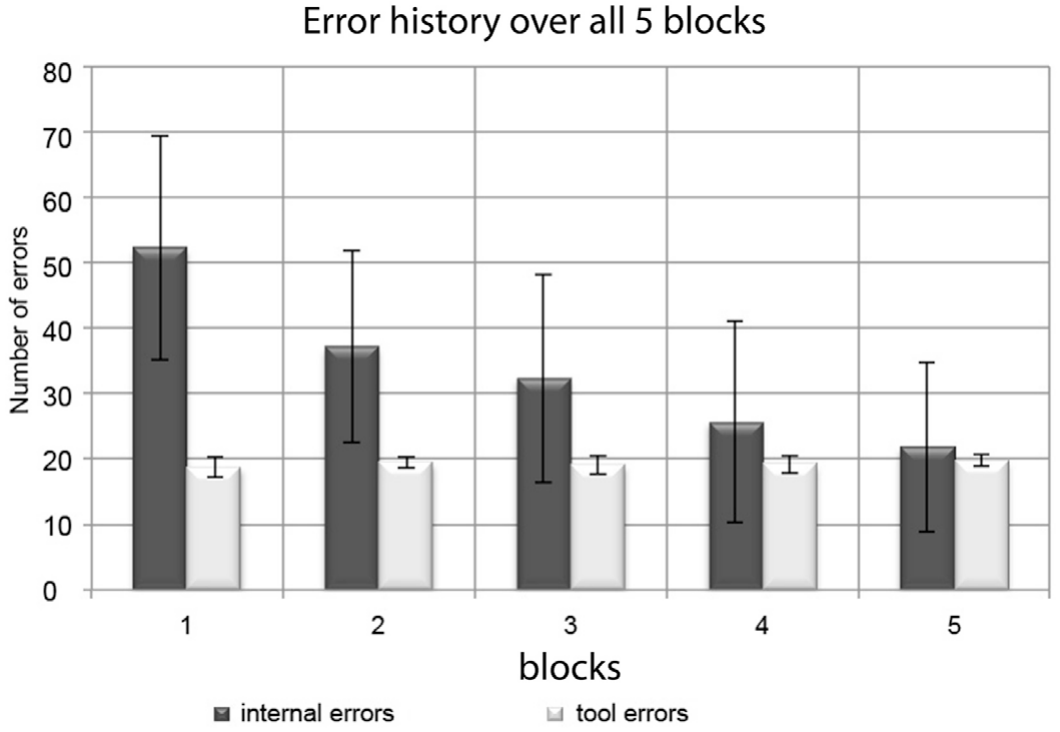
**Distribution of internal errors and tool errors over the experimental blocks**. Bars represent group mean values and error bars represent standard deviations. While the (experimentally induced) tool errors remained at a stable number of ~20 per block, the number of internal errors consistently decreased during the course of the experiment.

The absolute number of internal errors per session monotonically decreased from Session 1 to Session 5 (see Figure 3). A significant reduction from the beginning (Sessions 1 and 2; Mean errors per session ± *SD*: 45 ± 17) to the end of the experiment (Sessions 4 and 5; Mean errors per session ± *SD*: 24 ± 14) was confirmed by a paired *t*-test (*t* = 8.86; *p* < 0.001). Tool errors were introduced according to a predefined algorithm. Due to their programmed stochastic nature (see methods section), their temporal distribution varied only minimally, resulting in just short of 20 errors per session in all sessions.

Group means (± *SD*) of median response times from presentation of a target tone until a piano key was pressed are given in Table 1, separately for correct and false responses at the beginning and at the end (misses and multiple keypresses are excluded). Similarly, mean velocities of keypresses are listed separately for correct and false responses at the beginning and at the end in order to provide further evidence for the formation of an internal model, as indicated by the subjects' ability to match more closely the required target velocity at the end compared with at the beginning of the experiment.

**Table 1:** **Median Response times and Mean velocities of keypresses, divided into correct and false responses for the two learning stages**.

	**Median response times [ms]**		**Mean velocities [MIDI]**	
	**Beginning**	**End**	**Beginning**	**End**
Correct resp	896.4 (±71.8)	869.2 (±96.4)	43.79 (±5.71)	47.65 (±6.21)
False resp	1059.7 (±131.4)	1043.7 (±111.4)	42.32 (±5.56)	46.78 (±6.54)

There was no significant change in false response times from the beginning to the end [*t*_(18)_ = 0.60, *p* = 0.276]. However, correct response times significantly decreased [*t*_(18)_ = 1.78, *p* = 0.046]. MIDI velocities, indicating sound volume, approached the value given by the target tone (50). However, this change lacks statistical significance in both response categories (all *p* > 0.05).

### Electrophysiological data

**Figure 4:**
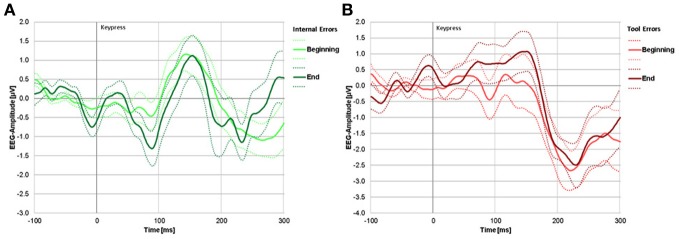
**(A)** Difference waves (error trials minus correct trials) for internal errors. The light green line represents internal errors during blocks 1 and 2 (beginning of experiment), the dark green line represents internal errors during blocks 4 and 5 (end of experiment). **(B)** Difference waves (error trials minus correct trials) for tool errors. The light red line represents tool errors during the beginning and dark red line represents tool errors during the end of the experiment. For both panels, solid lines represent group mean signal, dotted lines represent the standard error.

Mean difference waves (Error—correct response) are presented in Figure 4 for the two error types (IE and TE), averaged over participants and over the first two and the last two sessions. After artifact correction and elimination of unusable trials, a mean average across subjects of 33 (±6) TE trials were available at the beginning of the experiment and 36 (±3) at the end of the experiment. Internal error trials numbered on average 67 (±28) at the beginning and 38 (±25) at the end of the experiment.

A negative deflection due to internal errors can be seen at around 100 ms, but only at the end of the experiment (dark green line). A clear negative deflection due to tool errors can be seen only at around 230 ms. That peak is already apparent at the beginning of the experiment. Significance testing of signal deviation from zero at these peaks revealed no significant negativity at the beginning [*t*_(18)_ = −1.203, *p* = 0.249] or the end [*t*_(18)_ = 0.1670, *p* = 0.870] in response to tool errors within the time window of the ERN, but it did reveal significant negativity at the grand average peaks [*t*_(18)_ = −6.094, *p* < 0.001, and *t*_(18)_ = −2.357, *p* = 0.034, at the beginning and at the end, respectively] within the time window of the FRN for both learning stages.

In response to internal errors, there was a tendency for a negative deflection at the beginning and a clearly significant negative deflection at the end of the experiment within the time window of the ERN [*t*_(18)_ = −1.857, *p* = 0.085, and *t*_(18)_ = −3.167, *p* = 0.007, respectively]. Within the time window of the FRN, there was a just significant negative deflection at the beginning [*t*_(18)_ = −2.161, *p* = 0.049] but no significant negative deflection at the end of the experiment [*t*_(18)_ = −1.705, *p* = 0.110].

Analyzing the influence of learning stage and error-type on peak amplitudes, ANOVA revealed a significant main effect of error-type in the time interval related to the FRN [*F*_(18)_ = 12.256, *p* = 0.003] but no main effect of time [*F*_(18)_ = 0.109, *p* = 0.745] and no interaction of error type with time [*F*_(18)_ = 0.498, *p* = 0.489]. *Post-hoc t*-tests showed that tool errors are associated with stronger negative deflections during this interval than internal errors.

For the interval corresponding to ERN there was no main effect of either error type or time [*F*_(18)_ = 2.704, *p* = 0.117; and *F*_(18)_ = 0.628, *p* = 0.439, respectively], but an interaction between time and error type appeared [*F*_(18)_ = 21.323, *p* < 0.001].

**Figure 5:**
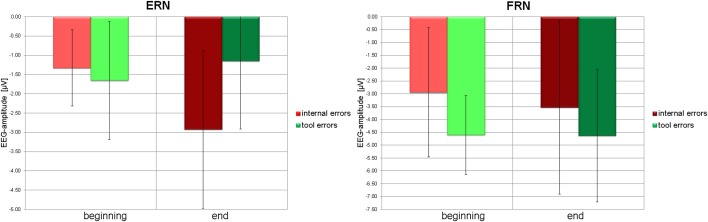
**Left panel:** Average amplitudes (filled bars) and standard deviations (error bars) of the ERN due to internal errors (red) and tool errors (green) at the beginning (light color) and at the end (dark color) of the experiment. **Right panel:** Average amplitudes (filled bars) and standard deviations (error bars) of the FRN due to internal errors (red) and tool errors (green) at the beginning (light color) and at the end (dark color) of the experiment.

*Post-hoc t*-tests revealed that ERN to internal errors is significantly smaller at the beginning than at the end of the experiment [*t*_(18)_ = 2.973, *p* = 0.008], whereas ERN to tool errors is insignificant in both learning stages. Negativity in the time window of the ERN even tends to decrease from beginning to end [*t*_(18) = −1.892_, *p* = 0.075]. Similarly, at the end of the experiment, ERN to internal errors is significantly stronger than ERN to tool errors [*t*_(18)_ = 3.295, *p* = 0.004], whereas no difference between error types exists at the beginning of the experiment [*t*_(18)_ = −0.743, *p* = 0.467]. See Figure 5 (left panel) for graphical illustrations of the differences.

On the other hand, there was a significant difference for FRN between tool errors and internal errors at the beginning of the experiment [*t*_(18)_ = −4.592, *p* = 0.000] and a tendency toward a difference at the end of the experiment [*t*_(18)_ = −1.671, *p* = 0.112]. No other tendencies or differences could be detected in pairwise comparisons of FRN amplitudes (all *p* > 0.15). See Figure 5 (right panel) for graphical illustrations of the differences.

As a prerequisite to interpretation of a change in ERN difference waves, we sought to make sure whether the EEG response to correct key presses changed from the beginning to the end of the experiment. We have tested, by means of a paired-samples *t*-test, whether the minimum EEG amplitude (time-locked to correct responses) in the time window of the ERN changes from the beginning to the end of the experiment. This is not the case [*T*_(18)_ = −1.482, *p* = 0.156].

**Figure 6:**
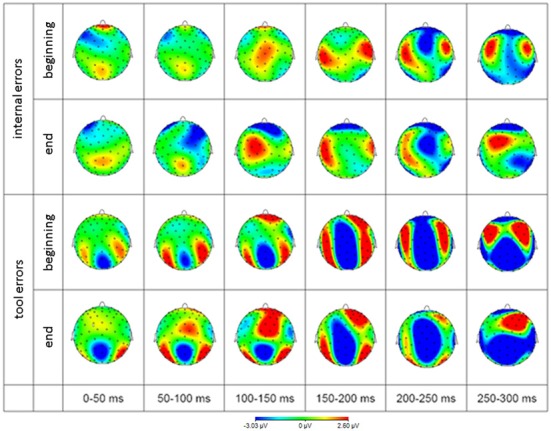
**Scalp distribution of EEG difference waves (error minus correct response) in response to internal errors (rows 1 and 2) and tool errors (rows 3 and 4) averaged over 50 ms time intervals**. Blue represents negativity and red represents positivity with respect to correct responses.

The scalp distribution of EEG activity is depicted in Figure 6 to give an impression about the extent of brain regions beyond the cluster of electrodes analyzed and described above.

## Discussion

The current study demonstrated that with formation of an internal model, the amplitude of the ERN, appearing in response to self-made errors, increases. On the other hand, the amplitude of the FRN, appearing regardless of the source of error, remains stable. Thus, ERN can be evoked, at least in part, by processes enabled by the internal model. Such a process may be the comparison of motor commands needed to reach a specific goal with motor commands actually issued. The present study, to the best of our knowledge, is the first to demonstrate the influence of internal model formation (audiomotor mapping) on ERN. In the following we discuss the specific findings of the study.

### Learning and formation of an internal model

On average, just over 50 wrong keypresses (i.e., ~25% of all presses) were committed by each subject at the beginning of the experiment (session 1). This shows that performance accuracy was already and clearly above chance in session 1. This was expected due to the systematic order of the keymapping. The results are consistent also with the assumption that the cognitive requirements on task performance were mainly served by the establishment of audio-motor associations. Audio-motor coupling forms an integral part of the internal model that is thought to be established during learning of a new musical instrument, as discussed in recent papers (e.g., Jäncke, 2012; Rodriguez-Fornells et al., 2012).

The traditional approach to the investigation of internal models has applied tasks involving visuomotor transformations such as required to generate movement trajectories. The current study therefore draws from this approach in order to develop a better understanding of how a musical instrument is learned. To describe changes in the processing of error information during the course of learning and to better understand the role of the ERN in this, a task was designed that mimicked the requirements of learning a keyboard instrument, allowed control for correctness of movements and ensured learning within one experimental session.

The number of internal errors at the end of the experiment was slightly more than 20 on average, and the number of tool errors remained constant at ~20 per session throughout the experiment, which is a common number of sweeps to use for within-subject averaging in order to derive amplitude measures for statistical group analysis.

Reaction times did not significantly change during the experiment, which is in line with the instruction to react regularly with a constant pace.

### Development of the ERN and FRN

The ERN and FRN represent difference waves, subtracting the EEG response to errors from the EEG response to correct responses. Therefore, to interpret the described changes in the ERN it is relevant to assess whether the correct response-related EEG remained stable. Only then, changes in ERN can be attributed to changed error processing. Since EEG reactions to correct responses have not changed significantly in the time window related to ERN, we interpret the change in difference waves to be a result of changed error processing.

While the ERN in response to internal errors and tool errors did not differ at the beginning of the experimental sessions, ERN due to internal errors increased and ERN due to tool errors tended to decrease from the beginning to the end of the experiment. This interaction effect was significant, showing that these two types of error increasingly diverged in the way that they modulated the ERN while the internal model was aquired over the course of the experimental sessions.

Our data thus provide new evidence that the ERN is based on information stored in an internal model that represents knowledge about mappings between actions and their consequences. Participants did not reliably reach the required goal at the beginning of our experiment, as evidenced by the high number of errors. But performance was clearly above chance even during the first session. This could be explained by the presence of implicit knowledge or expectations about the mapping between keys and tones (e.g., lower tones at the left, higher tones to the right) or by the possibility that the initial stages of learning and internal model acquisition could have already influenced performance during the first session. But the relatively high number of internal errors at the beginning of the experiment suggests that audiomotor coupling between the motor acts and the acoustic consequences was unreliable (i.e., error-prone) as would be expected prior to the formation of a correct internal model.

Without complete knowledge about the auditory-motor mapping, participants were unable to determine with absolute certainty whether a goal had been missed due to their own erroneous action or due to a incorrect performance of the tool itself. Consequently, there was no difference in ERN between these two types of error at the beginning of the experiment. In contrast, ERN to internal errors clearly increased in size at the end of the experiment when audiomotor coupling was known and the internal model established (Figure 4A). The ERN to internal errors was significantly larger than the ERN to tool errors at the end of the experiment, suggesting that the ERN is very likely modulated by information derived from the internal model.

That the ERN to internal error trials increased from the beginning to the end of the experiment is consistent with the assumption that the ERN reflects either expectancy violation or mismatch between required and actually performed motor plans. Both of these processes should be stronger at the end compared with the beginning of the experiment. Expectancy violation is not likely to be strong at the beginning of the experiment as the internal model on which basis clear expectancies would be formed has yet to be acquired. Similarly, participants do not know at the beginning which motor plan is required to reach a certain goal rendering it therefore unlikely that a mismatch between the required and actually performed motor plans would be detected and elicit EEG deflections at the beginning of the experiment. On the other hand, both expectancy violation and mismatch are likely at the end of the experiment on the basis of an established internal model. With each keypress, a sensory expectation will be formed and—in the case of tool errors—violated. Similarly, a motor act can be chosen upon perception of each acoustic goal that will, according to the internal model, generate the required tone. If during motor initiation or motor execution a wrong movement is performed, a mismatch between the required and the performed action would be detected and elicit an ERN.

It might be argued on the basis of the current results that the development of an ERN follows the detection of a mismatch between a required and performed motor act rather than a violated sensory expectancy. If violated sensory expectancy had caused the ERN, it should occur in the “tool error” trials, but it only occurred in “internal error” trials. This interpretation is in accordance with a recent conclusion of Potts et al. who scrutinized the commonalities of ERN, FRN and prediction errors (Potts et al., 2011). They found that the ERN consists of a single component usually elicited by behavioral error and that it also appears to be part of a more general prediction error system. In contrast, they found that the FRN appears to have several generators and is related to reward prediction errors. In the current investigation, the FRN developed at the beginning of the experiment at around 264 and 255 ms for internal and tool errors, respectively, and at the end of the experiment at 244 ms and 255 ms for internal and tool errors, respectively. The amplitudes of the FRN remained stable from the beginning to the end of the experiment in both error types. The fact that no significant influence of time can be detected on this error component leads to the conclusion that it is unrelated to the formation of an internal model. Thus, it is likely to rely on external feedback only, and no contribution of efference copies or other feedforward mechanisms could be detected using our method. This interpretation shares common ground with some forms of mismatch negativity (Alho, 1995; Näätänen, 1995; Näätänen et al., 2007): a stimulus, stored in sensory memory, is matched with a new stimulus, and upon mismatch, a negative deflection of the ERP is found around 150–250 ms after stimulus onset. This is also the case in our and many other error detection paradigms.

Interestingly, however, tool errors elicited a stronger FRN than internal errors with similar latencies. This gives way to the speculation that processing of external feedback information may be attenuated if internal errors have been committed. *Post-hoc* tests show that this is already the case at the beginning of the experiment when participants are not yet able to reliably distinguish internal from external errors. This leaves an open issue with the current data and might in fact show that even during the first two blocks (regarded to be the “beginning” of the experiment for the current purpose) an internal model is already present to a certain degree. This idea is supported by the behavioral data that already show a clear decrease in internal errors from block 1 to block 2. Thus, audiomotor coupling already occured during the first two blocks, although predictions about action consequences (and vice versa, decisions about actions needed to reach certain goals) cannot be made with certainty yet. In our view, the establishment of an internal model should be viewed, according to the present data, as a gradual process, possibly assigning certainties to feedforward predictions rather than providing a yes/no decision about action consequences. The FRN can therefore already be different at this early phase if we assume that it is influenced (attenuated) by uncertainty with regard to having pressed a wrong key.

It is possible that effects of response competition or motor implementation might have influenced the current data, but the experiment was designed to minimize such effects. There is no reason to assume that the number of conflicting responses might have increased during the course of experimentation as the choice of five response keys remained constant. In contrast, it might be assumed that conflict between these possible responses diminished during the course of the experiment as learning resulted in the exclusion of some response alternatives.

A limitation of the current study is that data had to be averaged over long time durations due to the fact that a relatively high number of trials is required for ERP analysis. This makes it impossible to describe gradual changes in the appearance of error-related EEG components. Thus, the distinction in the present paper between the “beginning” and “end” of the experiment must be seen in this context. During the “beginning” phase, audiomotor coupling and therefore associated, expectations of certain action consequences already form or are already present prior to testing. The current study is therefore unable to reveal why the FRN was already different between the two kinds of error at the beginning of the experiment before an internal model was well-established.

## Conclusion

The main finding of the current experiment is that an internal model which allows mapping of actions to sensory consequences significantly contributes to generation of an ERN over frontal midline areas. While this holds for ERN elicited by errors consisting of wrong motor acts, it does not apply for “errors” resulting from unpredictable changes in the environment. This experiment thus shows that the ERN results, at least in part, from the participant's realization that an actually performed action will not lead to the intended goal. On the other hand, the FRN, appearing over frontal midline areas around 250 ms after action initiation, is uninfluenced by the agent's capacity to map actions to their consequences. It follows that a detected mismatch between intended and actually realized action consequence (as analyzed by comparison of auditory events) is likely to be the main factor provoking this error component.

### Conflict of interest statement

The authors declare that the research was conducted in the absence of any commercial or financial relationships that could be construed as a potential conflict of interest.
